# Are the Long-Acting Intramuscular Formulations of Risperidone or Paliperidone Palmitate Associated with Post-Injection Delirium/Sedation Syndrome? An Assessment of Safety Databases

**DOI:** 10.2174/157488611794480070

**Published:** 2011-02

**Authors:** Larry Alphs, Srihari Gopal, Keith Karcher, Justine Kent, Jennifer Kern Sliwa, Stuart Kushner, Isaac Nuamah, Jaskaran Singh

**Affiliations:** 1 Ortho-McNeil Janssen Scientific Affairs, LLC, Titusville, New Jersey, USA; 2 Johnson & Johnson Pharmaceutical Research and Development, Titusville, New Jersey, USA

**Keywords:** Risperidone, Paliperidone Palmitate, Post-Injection Delirium/Sedation Syndrome.

## Abstract

Long-acting injectable (LAI) formulations of antipsychotics are valuable treatment alternatives for patients with psychotic disorders, and understanding their safe use is critical. Post-injection delirium/sedation syndrome (PDSS) has been reported following treatment with one atypical antipsychotic LAI. Clinical databases of risperidone LAI and paliperidone palmitate were explored to identify if cases of PDSS had been observed.

No cases of PDSS were identified in 15 completed trials of 3,164 subjects (approximately 115,000 injections) or the postmarketing safety database of risperidone LAI. Only one case of PDSS was identified among 10 completed trials (3,817 subjects, 33,906 injections) of paliperidone palmitate—that case having been reported in a patient randomized to treatment with placebo. Examination of these prospective databases finds no evidence that risperidone LAI and paliperidone palmitate are associated with PDSS and suggest that findings seen with another antipsychotic LAI are not generalizable.

## INTRODUCTION

Long-acting injectable (LAI) formulations of antipsychotic agents offer advantages to the use of orally administered agents in patients with schizophrenia who have difficulties with compliance and are at risk for relapse [1]. While these formulations are generally well-tolerated, a review by Citrome [2] and other reports [3-7] indicated a potential for patients to experience a constellation of neurologic symptoms following administration of one atypical antipsychotic LAI that are consistent with overdose and are referred to as post-injection delirium/sedation syndrome (PDSS). In a letter from the manufacturer [8], these events occur in <0.1% of injections and in approximately 2% of patients who received injections for up to 46 months. The time after injection to event ranges from soon after the injection to greater than 3 hours, with the risk being highest during the first hour [8]. According to the letter, the risk of an event is the same following each injection and, as so, cumulative (i.e., it increases with the number of injections) [8]. These analyses were performed to determine whether cases of PDSS or symptoms consistent with antipsychotic overdose have been reported for risperidone LAI and paliperidone palmitate.

## EVALUATION OF PDSS WITH RISPERIDONE LAI AND PALIPERIDONE PALMITATE

**Fig. (1):**
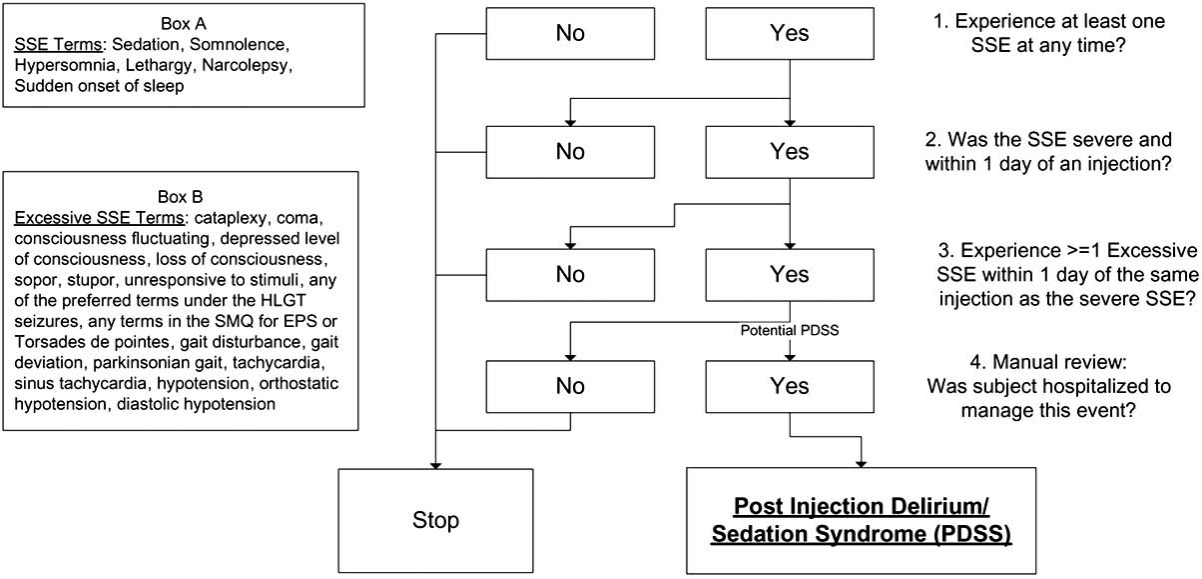
PDSS determination algorithm.

The clinical trial databases for 15 completed studies (3,164 subjects, ≈115,000 injections) and the postmarketing safety database for risperidone LAI, and from 10 completed clinical trials (3,817 subjects; 33,906 injections; 1,704.8 subject-years) of paliperidone palmitate were evaluated for reports of PDSS. A PDSS identification algorithm was created based upon literature descriptions of PDSS [5-8] methods and processes used in prior reports of antipsychotic LAI-related PDSS. The 4-part PDSS algorithm (Fig. **1**) included the following steps:

1. Development of a summary list of sedation/somnolence events of interest (SSEs) from subjects who experienced at least 1 event coded to MedDRA preferred terms of sedation, somnolence, hypersomnia, lethargy, narcolepsy, or sudden onset of sleep at any time;
2. Creation of a subset listing of subjects who experienced at least 1 severe SSE within 1 day of receiving an injection; and who experienced at least 1 of the following MedDRA events within 1 day of the injection associated with the severe SSE: cataplexy, coma, consciousness fluctuating, depressed level of consciousness, loss of consciousness, sopor, stupor, unresponsive to stimuli, any of the preferred terms under the MedDRA high level group term (HLGT) seizures, any of the terms within the standardized MedDRA query (SMQ) of extrapyramidal syndrome, gait disturbance, gait deviation, parkinsonian gait, tachycardia, sinus tachycardia, hypotension, orthostatic hypotension, diastolic hypotension, or any of the terms within the SMQ Torsades de Pointes; and finally;
3. Performance of a manual review of all subjects identified after the second step in the algorithm, limited to those who were hospitalized or required prolonged hospitalization to manage the severe sedation/somnolence event.

## PDSS FINDINGS WITH RISPERIDONE LAI AND PALIPERIDONE PALMITATE

Table **1**summarizes the incidences of treatment-emergent SSEs identified in steps 1 to 3 of the algorithm. Examination of the clinical trial and post-marketing risperidone databases revealed no cases of treatment-emergent SSEs that met the algorithm criteria for PDSS. Although 244 (8%) risperidone- and 8 (2%) placebo-treated subjects had at least 1 SSE, only 1 case (risperidone-treated) occurred within 1 day of the injection and it was not associated with concurrent excessive SSE symptoms and therefore did not meet the criteria for a PDSS event.

One case meeting all of the algorithm requirements for PDSS was identified in the review of the clinical trial paliperidone palmitate databases. This case occurred in a patient enrolled in a double-blind clinical trial who had been randomized to placebo treatment. The patient was a 39-year-old Asian female with a history of schizophrenia, mixed hyperlipidemia, obesity, and secondary parkinsonism. Within hours of her Study Day 1 administration of placebo, she experienced a generalized seizure followed by symptoms of drowsiness, urinary incontinence and post-ictal confusion (likely secondary to the seizure) and was hospitalized.

Upper 95% confidence limits for the probability of a PDSS event, given no events in 115,000 risperidone LAI injections and no events in 33,906 paliperidone palmitate injections are 0.003% and 0.01%, respectively.

## SUMMARY

No reports or signals of PDSS were identified in subjects treated with either risperidone LAI or paliperidone palmitate. Given the sample sizes explored, there is 95% confidence that the true probability of a PDSS event is less than 0.003% following risperidone LAI injections and less than 0.01% following paliperidone palmitate injections. PDSS has been proposed to occur as a result of systemic exposure to an LAI with high blood/plasma solubility. Neither risperidone LAI or paliperidone palmitate have high water solubility. Risperidone LAI is an aqueous suspension wherein the active drug substance is encapsulated in a microsphere of polylactide-co-glycolide polymer. After injection, a small amount of risperidone (<1%) is released by diffusion within 24 hours. The remaining drug is gradually released as the microsphere copolymer is hydrolyzed and result in a low and steady release of risperidone over a period of several weeks, with the majority being released during weeks 4 to 6 following injection [9, 10]. Paliperidone palmitate dissolves slowly after intramuscular injection and is hydrolyzed to paliperidone, producing sustained therapeutic plasma concentrations with administration of two initiation doses (administered one week apart) followed by once-monthly maintenance doses [11].

In summary, these results suggest that risperidone LAI and paliperidone palmitate do not have measurable risk for post-injection delirium/sedation syndrome.

## Figures and Tables

**Table 1:** Incidence of SSE (Any, Severe and Excessive) Identified in the Paliperidone Palmitate and Risperidone LAI Clinical Trial Databases

Algorithm Step	Placebo	Paliperidone Palmitate	Risperidone LAI
**Step 1. Any Sedation/Somnolence Event (SSE)**	***Number of Subjects with ≥1 SSE/Total Subjects (%)***		
Paliperidone palmitate studies	19/713 (3%)	95/3,817 (3%)	28/1,199 (2%)
Risperidone LAI studies	8/323 (2%)	---	244/3,164 (8%)
**Step 2. Severe SSE within 1 day of injection **	***Severe SSE Events/Total Injections***		
Paliperidone palmitate studies	1/2,370	2/33,906	0/13,497
Risperidone LAI studies	0/3,713	---	1/115,000
**Step 3. Satisfied Step 2 and had an Excessive SSE within 1 day of the same injection as in Step 2**	***PDSS Events/Total Injections***		
Paliperidone palmitate studies	1/2,370	0/33,906	0/13,497
Risperidone LAI studies	0/3,713	---	0/115,000
